# Greater Performance of Exotic *Elodea nuttallii* in Response to Water Level May Make It a Better Invader Than Exotic *Egeria densa* During Winter and Spring

**DOI:** 10.3389/fpls.2019.00144

**Published:** 2019-02-25

**Authors:** Yalin Wang, Xiuwen Chen, Junchu Liu, Yaping Hong, Qiankun He, Dan Yu, Chunhua Liu, Huanjiang Dingshanbayi

**Affiliations:** The National Field Station of Freshwater Ecosystem of Liangzi Lake, College of Life Sciences, Wuhan University, Wuhan, China

**Keywords:** invasive, submerged, water depth, overwinter, morphological, photosynthesis

## Abstract

The strategy of producing rapid initial growth and establishing early in the growing season is important, and it is employed by invasive macrophytes. *Elodea nuttallii* and *Egeria densa*, two Hydrocharitaceae species, became weeds after invading many countries in recent years. Comparative studies on their invasive traits in relation to native species during winter and spring are limited. In the present study, we compared the growth performance of these two exotic species with a perennial native species, *Potamogeton maackianus*, in different water depths (1, 2, and 3 m) during winter (January and February) and spring (March and April). Three morphological traits (shoot number, root number and shoot length), total biomass, relative growth rate (RGR) and two physiological photosynthetic traits (total chlorophyll content and the maximum quantum yield of PSII [*F*v/*F*m]) were measured for each macrophyte. All three species could overwinter as entirely leafy plants. Biomass, RGR, morphological traits and physiological traits were all different among species. However, water depths had a significant effect only on morphological traits. At all water depths, *E. nuttallii* had significantly higher values for morphological traits, total biomass and RGR than *P. maackianus*, while *E. densa* had significantly fewer roots and a lower total chlorophyll content than *P. maackianus*. Except for *F*v/*F*m at a 3 m water depth, morphological and physiological photosynthetic traits, biomass and RGR of *E. nuttallii* were significantly higher than those of *E. densa.* In addition, a large number of adventitious roots developed from *E. nuttallii* but not from the other two species. These results indicate that the advantages of *E. nuttallii* to grow in winter and spring may make it more prone to expansion than *E. densa* in China.

## Introduction

Currently, with the process of economic globalization and trade liberalization, many species are imported in places that are out of their indigenous geographical ranges, and some of them develop into invasive species (Richardson et al., 2000). With its negative effects, biological invasion of exotic species has become a universal ecological problem (Mack et al., 2000). Compared to controlling outbreaks of invaders, prevention is more feasible and economical (Waage and Reaser, 2001). Therefore, it is important to predict the potential invasiveness of species (Rejmánek and Richardson, 1996; Barrat-segretain et al., 2002), which can help us set priorities for the control of introduced invasive species (Rejmánek and Richardson, 1996).

In freshwater ecosystems, invasive plants are alien species that possess advantages for proliferation (produce large numbers of sexual and/or vegetative propagules), dispersal (multiple means and long-distance dispersal) and rapid colonization (Richardson et al., 2000; Kercher and Zedler, 2004). Numerous studies have considered invasive aquatic plants with regards to their growth, regeneration capacity, photosynthesis traits, genetic, reproductive, overwintering strategies and management (Lui et al., 2005; Hussner, 2009; Zhang et al., 2010; Wersal and Madsen, 2011; You et al., 2014; Liu et al., 2016; Hussner et al., 2017). Water depth could affect morphological traits, photosynthetic traits, reproduction and distribution of native or exotic aquatic macrophytes (Chambers and Kaiff, 1985; Strand and Weisner, 2001; Xiao et al., 2010; Fan et al., 2015; Liu et al., 2016; Xu et al., 2016; Han et al., 2018; Li et al., 2018; Zhao et al., 2018). On the other hand, overwintering is important for the invasion of exotic species. The strategy of invasive macrophytes to produce rapid initial growth and establish early in the growing season may increase their competitive ability and allow them to successfully replace native species (Nichols and Shaw, 1986). In addition, the response of submerged macrophytes to the environment might differ depending on the timing of environmental changes and the growth phases of plants (Cao et al., 2015). However, the influence of water depth on the growth of invasive submerged species during the early growth season has not been well elucidated.

*Elodea nuttallii* (Planch.) H. St John is a submerged macrophyte and is native to temperate North America (Cook and Urmi-König, 1985). It has been defined as an invasive species and is now receiving increasing attention for its rapid and lasting invasion of many freshwater habitats throughout Europe, Asia and Australia (Kunii, 1981, 1982, 1984; Barrat-segretain et al., 2002; Zehnsdorf et al., 2015). Another submerged macrophyte, *Egeria densa* (Planch.), which is native of South America, became a weed after it was introduced to North America, Europe and New Zealand (Cook and Urmi-König, 1984; Champion and Tanner, 2000; Gassmann et al., 2006). It can form monodominant and dense stands which have negative effects on native water ecosystems (Yarrow et al., 2009; Fujiwara et al., 2016). *E. nuttallii* and *E. densa* are both species that can tolerate and survive cold temperature, even surviving under ice cover (Kunii, 1984; Yarrow et al., 2009). Moreover, both species can tolerate a wide range of water depths (Vöge, 1994; Carrillo et al., 2006). *E. nuttallii* was introduced to China approximately 30 years ago (Xu et al., 2007). Previous studies considered the influence of *E. nuttallii* on the native ecosystems of China (Zhang et al., 1999) and found that this species had begun to invade the local aquatic community of the East Taihu Lake (Gu et al., 2005) and expanded in other sites in recent years (Yu et al., 2018). *E. densa* was introduced in China as an ornamental plant in recent years, but it has escaped and naturalized in fields, with a limited scope (Yu et al., 2018).

The objective of this study was to compare the growth under various water depths (1, 2, and 3 m) of these two species during winter and spring to that of native species *Potamogeton maackianus*, which is a primary submerged macrophyte in the Yangtze Plain of China that can grow in the winter (Ni, 2001; Su et al., 2019). We hypothesized that (1) not both of exotic species may have advantages in growth traits over native species and (2) water depths would influence the growth of all three macrophytes during winter and spring, and the effects of water depths were expected to be different for native and exotic species.

## Materials and Methods

### Plant Materials and Study Sites

This study was conducted at The National Field Station of Freshwater Ecosystem of Liangzi Lake, Wuhan University, China (30°5′–30°18′N, 114°21′–114°39′E). In early November 2013, all samples of two exotic species (*E. nuttallii* and *E. densa*) and one native species, *P. maackianus* were collected from the field station. We chose 37 apical shoots of each plant for the experiment, which were collected from parent individuals with an equal length of 15 cm. For each species, we recorded the fresh weight of all shoots, and we used the fresh and dry (after drying at 70°C for 72 h) weights of 16 apical shoots for the transformation. The mean dry weights of the shoots of *E. nuttallii*, *E. densa*, and *P. maackianus* were 0.024 ± 0.001, 0.116 ± 0.009, and 0.066 ± 0.005 g (mean ± SD), respectively.

### Experimental Design

**FIGURE 1:**
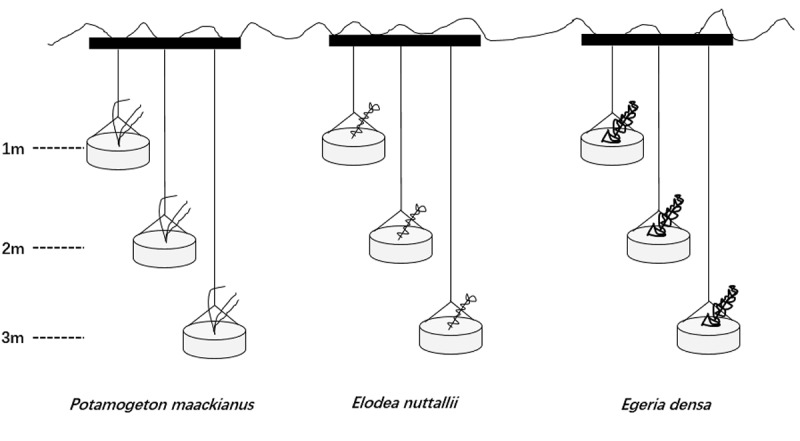
Design of the experiments showing three of the 9 containers in which the three species were planted.

We set three water depths (1, 2, and 3 m) in this experiment because the actual mean water depth is 3.0 m in Liangzi Lake (Xu et al., 2018). The experiment was conducted in outdoor cement pools (4 m × 4 m × 4 m; length × width × height) filled with lake water [TN 0.71 ± 0.01 mg L^-1^,TP 0.04 ± 0.01 mg L^-1^, measured with a YSI Professional Plus water quality meter (YSI Inc., Yellow Springs, OH, United States)]. Seven cement pools were used and one pool represented a replicate. We placed three steel tubes (>4 m; length) on each cement pool at the same intervals and hung three rubber buckets (25 cm × 15 cm; diameter × depth) on each steel tube with the same intervals (Figure 1). One apical shoot was planted in a rubber bucket filled with homogeneous mud sediment [TN 0.74 mg g^-1^, TP 0.03 mg g^-1^, measured with IL500 Automatic Analyzer (Hach Corp., Loveland, CO, United States)] from Liangzi Lake and placed at the water level of 0.5 m for a month to root and acclimatize. A month later, we used ropes to control three water depths (1, 2, and 3 m) (Figure 1).

A total of 21 individuals of each species were used. During the experiment, lake water was added to cement pools twice a week to compensate for evaporation loss. The water temperature and light intensity were recorded twice a week, and the average daily temperature of each month from January to April was 7.22 ± 0.5, 7.12 ± 1.96, 10.57 ± 1.9, and 16.74 ± 2.9°C (mean ± 1SE); photosynthetically active radiation (PAR) at noon at different water depths (1, 2, and 3 m) was 108.6 ± 90.1, 65.8 ± 57.0, and 14.8 ± 13.4 μmol m^-2^ s^-1^, respectively (means ± 1SE). The experiment lasted 5 months (from 22 November 2013 to 22 April 2014, including a month for acclimation).

### Trait Measurement

The shoot number, root number (rooted in sediment), shoot length and two physiological photosynthetic traits (total chlorophyll content, maximum quantum yield of PSII [*F*v/*F*m]) were measured for each macrophyte. Adventitious roots (water roots) number of *E. nuttallii* were also recorded. All biomass was obtained after drying plants in an oven at 70°C for 72 h. Relative growth rate (RGR) was calculated as RGR = (ln*w*_2_ – ln*w*_1_)/(*t*_2_ – *t*_1_), where *w*_1_ and *w*_2_ stand for initial and final dry weight, respectively, and (*t*_2_ – *t*_1_) represents the experimental duration of each species from 22 November 2013 to 22 April 2014. A portion of 0.1 g of leaves (fresh weight) obtained from each plant was used for the measurement of chlorophyll content. Plant material was ground to a fine powder, and then extracted with 95% ethanol. The total chlorophyll content (the summed values of chlorophyll a and b) was determined by spectrophotometer, according to Lichtenthaler (1987). Chlorophyll fluorescence on the fully developed, healthy leaves from the top was measured after harvesting. Before measurements, leaves were covered for at least 20 min to allow for dark adaptation, then the maximum (*F*m) and the minimum (*F*o) fluorescence yields were measured with the saturation pulse method (Schreiber et al., 2000), with a portable chlorophyll fluorometer (DIVING-PAM, Walz, Effeltrich, Germany). Variable fluorescence (*F*v) was calculated as *F*m–*F*o; consequently, the maximum quantum yield of PSII (*F*v/*F*m) was obtained.

### Statistical Analysis

Differences in growth traits were analyzed first by analysis of covariance (ANCOVA) with initial biomass as the covariate. Initial biomass did not significantly affect any growth traits (all *P* > 0.05). Then, two-way ANOVA with water depths and species as the main factors was performed to determine the main effects and interactive effects on all growth traits. If a significant treatment effect was detected, *post hoc* pairwise comparisons of means were made to examine differences between treatments using studentized Tukey’s HSD for multiple comparisons. One-way ANOVA was performed to determine the effects of water depth on the water root number of *E. nuttallii.* Data were log-transformed (shoot and root number, total biomass and RGR) or square root-transformed (shoot length) to ensure normality of residuals and homogeneity of variances, and homogeneity was tested using Levene’s test. All data analyses were conducted using SPSS 18.0 (SPSS, Chicago, IL, United States).

## Results

**FIGURE 2:**
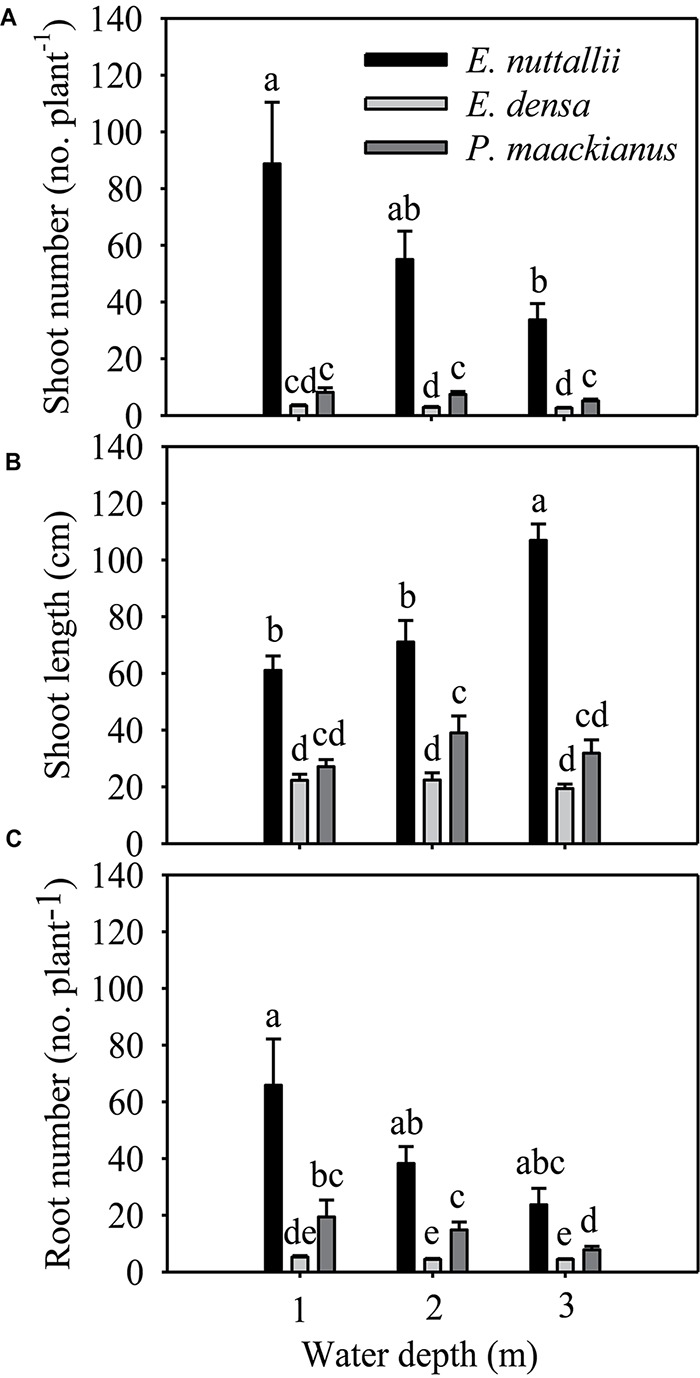
Changes in the morphological traits (shoot number **A**, shoot length **B**, and root number **C**) of the three macrophytes, data are presented as the means ± 1SE (*n* = 7). Means with different small letters are significantly different at *P* < 0.05 among different treatments.

All morphological traits were significantly different between species and water treatments (Table 1). All morphological traits (shoot number, shoot length and root number) of *E. nuttallii* were significantly higher than those of *E. densa* and *P. maackianus* at all water depths (Figure 2A–C). The shoot number of *P. maackianus* at 2 and 3 m depths and the shoot length of *P. maackianus* at the 2 m depth were significantly higher than those of *E. densa* (Figure 2A,B). The root number of *E. densa* was significantly lower than that of *P. maackianus* at all depths (Figure 2C). With increasing water depth, the shoot number and shoot length of *E. nuttallii* decreased and increased, respectively, while depth had no significant effect on the shoot traits of *E. densa* and *P. maackianus* (Figure 2A,B). Water depth had no significant effect on the root number of *E. nuttallii* and *E. densa*, while the root number of *P. maackianus* at the 3 m depth was significantly lower than that of plants at the 1 and 2 m depths (Figure 2C). Depth had no significant effect on the water root number (*F* = 0.192, *P* = 0.827) of *E. nuttallii*, and the mean number of water roots was 54.71 ± 9.85 per plant among all three water depths.

**Table 1:** *F* values and *P* values for species and water depth treatments for shoot number, root number, shoot length, total biomass, RGR, total chlorophyll content and *F*v/*F*m by two-way ANOVA (*n* = 7).

Traits	Species (S)		Water depth (W)		S × W	
	*F*	*P*	*F*	*P*	*F*	*P*
Shoot number^a^	197.671	**0.000**	5.874	**0.005**	0.682	0.608
Root number^a^	100.195	**0.000**	6.703	**0.003**	1.154	0.341
Shoot length^s^	129.582	**0.000**	5.136	**0.009**	6.457	**0.000**
Total biomass^a^	56.852	**0.000**	2.190	0.122	0.038	0.997
RGR^a^	55.168	**0.000**	2.615	0.083	0.143	0.965
Total chlorophyll content	24.578	**0.000**	0.327	0.723	1.475	0.223
*F*v/*F*m	17.789	**0.000**	1.866	0.165	0.532	0.713


*^a^Log transformed, ^s^Square root transformed. Bold type is used for significant differences (P < 0.05).*

**FIGURE 3:**
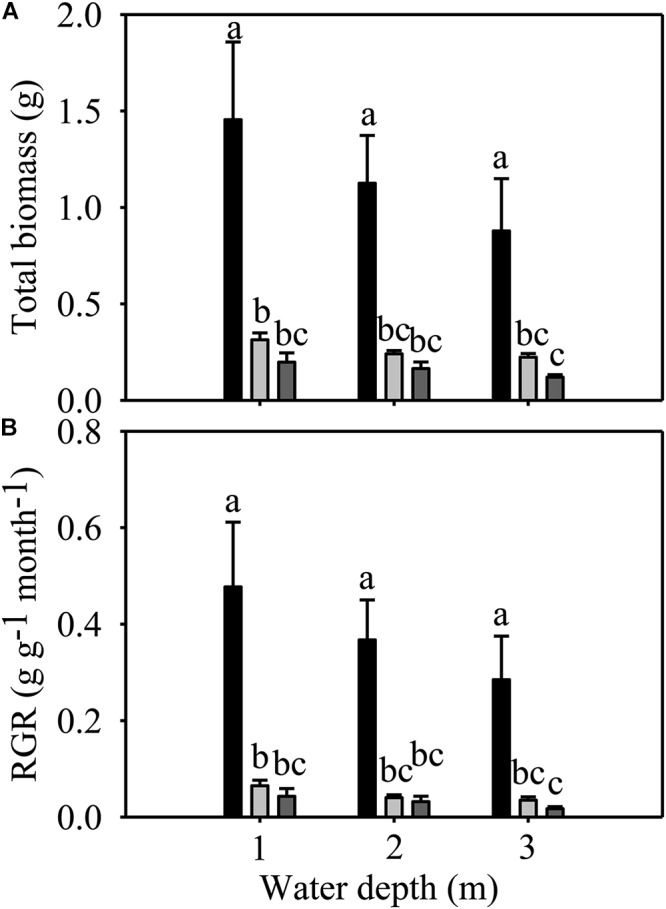
Changes in the total biomass **(A)** and RGR **(B)** of three macrophytes. The data are presented as the mean ± 1SE (*n* = 7). Means with different small letters are significantly different at *P* < 0.05 among different treatments.

Total biomass and RGR were significantly different among species but not among different water depths (Table 1). The total biomass and RGR of *E. nuttallii* were significantly higher than those of *E. densa* and *P. maackianus* at all water depths (Figure 3A,B). There were no significant differences in total biomass and RGR between *E. densa* and *P. maackianus* (Figure 3A,B).

**FIGURE 4:**
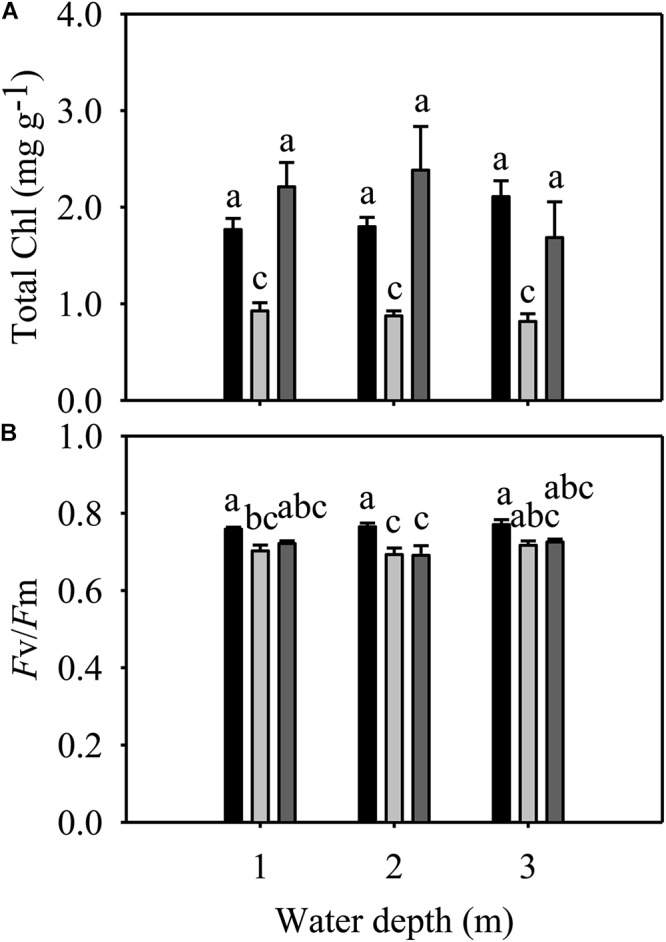
Changes in the total chlorophyll content **(A)** and *F*v/*F*m **(B)** of three macrophytes. The data presented are the mean ± 1SE (*n* = 7). Means with different small letters are significantly different at *P* < 0.05 among different treatments.

Total Chl and *F*v/*F*m were significantly different among species but not among different water depth treatments (Table 1). At all three water depths, the total Chl of *E. nuttallii* was not significantly different from that of *P. maackianus*, while the total Chl of *E. densa* was significantly lower than that of the other two species (Figure 4A). At the 1 m depth, both *F*v/*F*m of *E. nuttallii* and *E. densa* were similar to *P. maackianus*, while *F*v/*F*m of *E. nuttallii* was significantly higher than that of *E. densa* (Figure 4B). At the 2 m depth, *E. densa* and *P. maackianus* had no difference in *F*v/*F*m, while both values were significantly lower than that of *E. nuttallii* (Figure 4B). The difference in *F*v/*F*m among the three species disappeared when plants were grown at a depth of 3 m (Figure 4B).

## Discussion

Previous studies revealed that some exotic aquatic plants survival increased with increasing winter temperatures (Hussner and Lösch, 2005; You et al., 2014; Liu et al., 2016), which may be responsible for facilitating their invasion. Our results revealed that both exotic macrophytes could overwinter as entire plants. Compared with invasive species that cannot overwinter, overwintering ability may aid these species in invasion.

The shoot number, shoot length, root number, total biomass and RGR of *E. nuttallii* were significantly higher than those of *E. densa* and *P. maackianus* at all water depths (Figure 2A–C, 3A,B). This suggests that the recovery of *E. nuttallii* happens earlier than the other two species, consistent with the findings of *E. nuttallii* compared with *E. canadensis* (Ozimek et al., 1993). This may be due to the extension of the growth season of *E. nuttallii*, beginning in winter with the cumulative daily water temperatures over 4°C (Kunii, 1981); the critical temperature for its active growth lies between 8.2 and 12.0°C (Kunii, 1982), which is lower than the temperature for active growth of many other submerged plants (Ozimek et al., 1993). It was also found that *E. nuttallii* grows well and has a competitive advantage over native species at low water temperatures in Japan (Kunii, 1981, 1982, 1984). The strategy adopted by invasive macrophytes to produce rapid initial growth and establish early in the growing season is a much less conservative life strategy than that of the species that grow and reproduce until midsummer (Nichols and Shaw, 1986); thus, our results suggested that the early development of *E. nuttallii* might be another important means to promote its colonization. A similar phenomenon of the winter growth of *E. nuttallii* has been reported in its related species *E. canadensis* (Haag, 1979). *E. densa* did not show advanced winter growth, compared to *E. nuttallii* and the indigenous *P. maackianus*, in the present study. The winter growth of *E. densa* is slow and this species tends to store starch for subsequent spring growth (Yarrow et al., 2009). It can recover from winter senescence and quickly reinvade water bodies through energy stored in basal stems and the root crown (Pennington and Sytsma, 2009; Cabrera et al., 2013), but the optimal temperature range for its active growth is 14 to 25°C (Pennington, 2008). However, the water temperature was only up to 14°C in late April, which could explain why *E. densa* grew slowly and did not recover during the experimental period.

Once the invasion of exotic macrophytes has taken place, subsequent colony growth proceeds primarily by stem fragmentation (Hofstra et al., 1999), owing to clonal fragmentation being one of the essential means of duplication and dispersal of aquatic plants (Barrat-segretain, 1996). The three macrophytes in our study mainly reproduce vegetatively through clonal growth of stolons or fragments (Cook and Urmi-König, 1984; Li et al., 2004; Nagasaka, 2004). Therefore, the number of propagules could be reflected by their shoot number and shoot length. In our study, the shoot number and shoot length of *E. nuttallii* were significantly higher than that of *P. maackianus*, whereas the reverse was true for *E. densa* under most treatment conditions (Figure 2A,B), which might aid *E. nuttallii* and inhibit *E. densa* in occupying niches early in the spring. The number of invaders (propagule pressure) is one of the traits that influence the ability of species to invade new communities (Lonsdale, 1999; Xie et al., 2013). Furthermore, *E. nuttallii* may produce a canopy shading out and inhibiting other species due to its shoot length (Barrat-segretain and Elger, 2004); a similar study suggested that *E. nuttallii* invests more in shoot elongation than *E. canadensis* and thus has a greater advantage for light capture (Szabó et al., 2019). We suggested that the earlier the start of the vegetative period, the higher the capability for vegetative reproduction, and a greater shoot length might contribute to *E. nuttallii* being more invasive than *E. densa* during winter and spring.

The roots of submerged plants could maintain many of the functions of terrestrial roots, such as the absorption and transportation of nutrients and water and the supply of shoots with anchorage and phytohormones (Agami and Waisel, 1986; Hussner et al., 2009). The root number of *E. densa* was significantly lower than that of the other two macrophytes in all treatments, while that of *E. nuttallii* was the highest among the three species at all water depths (Figure 2C). Since this species can absorb and accumulate large quantities of nitrogen through its roots (Ozimek et al., 1993), the greater amount of roots of *E. nuttallii* may result in an advantage in nitrogen absorption over the other two species. A previous study also found that another invasive species, *E. canadensis*, allocated more biomass to roots during adverse environmental conditions (heat wave) to absorb more nutrients (Cao et al., 2015). Furthermore, adventitious roots were of greater importance in P-storage under high resource availabilities (Garbey et al., 2004). The development of adventitious roots may aid the survival through the uptake of water column nutrients, which may be a mechanism for survival during adverse conditions, a means of long distance dispersal of fragments, or it may offer a competitive advantage over species that rely on sediment nutrients (Wersal and Madsen, 2011). Moreover, the development of adventitious roots may contribute to the ability to spread and colonize in local habitats or in new habitats once fragments occur, because shoots are very often broken and fragmented by animals and water flow (Barrat-segretain, 1996), in plants that possess higher survival rates (Barrat-segretain et al., 2002). Adventitious roots (water roots) developed from shoots of *E. nuttallii* in our experiment, as observed by previous studies, were located at nodes of lateral branches (Ozimek et al., 1993; Barrat-segretain, 2005). Therefore, the development of more roots and adventitious roots in *E. nuttallii* may contribute to its rapid growth and distribution.

Since the conditions of light and substrate availability change and waves may occur with increasing water depth (Duarte and Kalff, 1990), and light intensity underwater decreases significantly (Zhu et al., 2012), water depth has an important influence on the growth, reproduction and distribution of aquatic macrophytes (Chambers and Kaiff, 1985; Xiao et al., 2010; Zhu et al., 2012; Fan et al., 2015; Li et al., 2018; Zhao et al., 2018). Contrary to our hypothesis, our results showed that water depth had less influence on most traits of *E. nuttallii* and *P. maackianus*, except that deeper water (3 m) decreased the shoot number of *E. nuttallii* and root number of *P. maackianus*, and increased the shoot length of *E. nuttallii*. Similar results were reported for other species, also including *P. maackianus* (Strand and Weisner, 2001; Yang et al., 2004; Zhu et al., 2012; Fan et al., 2015). In addition, in this study, water depth had no influence on any traits of *E. densa* (Figure 2–4). Previous studies found that these three species could tolerate a wide range of water depths and adapt well under low light conditions (Barko and Smart, 1981; Vöge, 1994; Ni, 2001; Carrillo et al., 2006), which probably accounts for the indistinct differences in their growth among different water depths in our study. In addition, plant growth was not essentially sensitive to light intensity at cool temperatures (Rajan et al., 1973). The *F*v/*F*m ratio is normally within the range of 0.75–0.85, and the decline in this ratio indicates photoinhibitory damage caused by environmental stresses (Bolhár-Nordenkampf et al., 1989). For example, water depth (≥3 m) severely reduced the *F*v/*F*m of the submerged plants *Vallisneria natans*, indicating that flooding treatments imposed severe stress on the fitness of this species (Han et al., 2018), or shallow water decreased *F*v/*F*m of the submerged plants *Ottelia acuminata* because of the strong light (Zhao et al., 2018). However, water depth had no significant effects on the *F*v/*F*m of the three macrophytes in our study (Figure 4B). Similarly, there was no significant change in the total Chl content of the three species among different water depth treatments (Figure 4A). The maintenance of the photosynthetic pigment concentration (Chl concentration) explains the stability of *F*v/*F*m (Meneguelli-Souza et al., 2016). These results suggested that 2 m or 3 m water depths did not cause damage to these three macrophytes, because they can tolerate a wide range of water depths (Vöge, 1994; Carrillo et al., 2006; Zhu et al., 2012; Li et al., 2013). Contrary to our second hypothesis, the growth of these three macrophytes was less influenced by water depth.

## Conclusion

All three species could overwinter as whole leafy plants. The growth and photosynthesis of the three species were less influenced by water depths. The results verified our first hypothesis that not both exotic species showed advantages over the native species. *E. nuttallii* presented superior advantages over others observed in its considerable growth and shoot increase (such as the larger RGR, more shoots and roots, and higher shoot length) in late winter and spring, which might help it to establish in its niche early and express its invasive traits, thus favoring its expansion and potential outbreak. By contrast, *E. densa* was disadvantaged compared with the other two macrophytes because of its weak growth and photosynthetic rate in winter and early spring.

## Author Contributions

YW, DY, and CL designed the research and executed the research project. YW, XC, JL, YH and QH collected the field data. YW and CH analyzed the data and wrote the manuscript. HD revised the paper critically.

## Conflict of Interest Statement

The authors declare that the research was conducted in the absence of any commercial or financial relationships that could be construed as a potential conflict of interest. The handling Editor is currently organizing a Research Topic with one of the authors CL, and confirms the absence of any other collaboration.
